# Interactions of bacteriophage T4 adhesin with selected lipopolysaccharides studied using atomic force microscopy

**DOI:** 10.1038/s41598-018-29383-w

**Published:** 2018-07-19

**Authors:** Ewa Brzozowska, Adam Leśniewski, Sławomir Sęk, Ralph Wieneke, Robert Tampé, Sabina Górska, Martin Jönsson-Niedziółka, Joanna Niedziółka-Jönsson

**Affiliations:** 1 Hirszfeld Institute of Immunology and Experimental Therapy, Polish Academy of Sciences, 12R. Weigl, 53-114 Wrocław, Poland; 2 Institute of Physical Chemistry, Polish Academy of Sciences, 44/52 Kasprzaka, 01-224 Warszawa, Poland; 3 Biological and Chemical Research Centre, University of Warsaw, 101 Żwirki i Wigury, 02-089 Warszawa, Poland; 4 Institute of Biochemistry, Biocenter, Goethe University Frankfurt, Max-von-Laue-Str. 9, 60438 Frankfurt am Main, Germany

## Abstract

The interaction between the T4 bacteriophage gp37 adhesin and the bacterial lipopolysaccharide (LPS) is a well-studied system, however, the affinity and strength of the interaction haven’t been analyzed so far. Here, we use atomic force microscopy to determine the strength of the interaction between the adhesin and its receptor, namely LPS taken from a wild strain of *E*. *coli* B. As negative controls we used LPSs of *E*. *coli* O111:B and *Hafnia alvei*. To study the interaction an AFM tip modified with the gp37 adhesin was used to scan surfaces of mica covered with one of the three different LPSs. Using the correlation between the surface topography images and the tip-surface interaction we could verify the binding between the specific LPS and the tip in contrast to the very weak interaction between the tip and the non-binding LPSs. Using force spectroscopy we could then measure the binding strength by pulling on the AFM tip until it lifted off from the surface. The force necessary to break the interaction between gp37 and LPS from *E*. *coli* B, LPS from *E*. *coli* O111:B and LPS from *H*. *alvei* were measured to be 70 ± 29 pN, 46 ± 13 pN and 45 ± 14 pN, respectively. The latter values are likely partially due to non-specific interaction between the gp37 and the solid surface, as LPS from *E*. *coli* O111:B and LPS from *H*. *alvei* have been shown to not bind to gp37, which is confirmed by the low correlation between binding and topography for these samples.

## Introduction

Bacteriophages are viruses that infect bacteria^1^. The first step of bacterial infection is the recognition of an outer membrane receptor. The bacteriophages’ ability for specific recognition of bacteria is used in many fields of science and technology such as bacteria identification by the Phage typing method^2,3^ or as bacteria detection using phage-based sensors^4–7^. The bacteriophage T4 belongs to the *Myoviridae* family and is a ubiquitous microorganism found in the environment as well as in the microbiomes of eukaryotic organisms^8^. T4 is one of the most studied lytic phages that infects only limited strains of *Escherichia* and *Shigella* and is a universal research model in microbiology to study the mechanisms of many biological processes, including bacteriophage infection^9^. This bacteriophage was originally isolated on *Escherichia coli* B wild-type strain. During the adsorption process, T4 uses long and short tail fibers. First, at least three of its six long tail fibers (LTF) reversibly bind to the host cell receptors. Next, the short tail fibers (STF) bind irreversibly to the receptors^10^. The adsorption specificity of T4 is determined by the tip of the adhesion protein called adhesin gp37 coded by gene 37. Gp37 is localized in the distal part of the each LTF which are symmetrically anchored around the baseplate^11^.

T4 bacteriophage recognizes either *E*. *coli* B type lipopolysaccharide (LPS) or OmpC protein with identical efficiency^12^. LPS is the main surface component of Gram-negative bacteria and can be grouped into a smooth type (S-LPS) or rough type of LPS (R-LPS). S-LPS consists of three regions: an O-specific polysaccharide, a core oligosaccharide and the lipid A. The lipid A anchors the molecule in the outer membrane. The R-LPS is devoid of the O-specific chain and thus is structurally a lipooligosaccharide (LOS)^13^. The LPS of *E coli* B belongs to the R-type of LPS (LOS) and it is very specifically bonded by gp37 adhesin of T4 bacteriophage^5^.

In this paper we utilize AFM measurements to determine the interactions between T4 gp37 adhesin with three different LPS – one which is a binding target for gp37 (LPS from *E*. *coli* B) and two that are non-binding (LPSs from *E*. *coli* O111:B and *Hafnia alvei* PCM 1189)^5^. AFM in the PicoTREC mode has been used to localize the LPSs that interacts with the gp37 protein. The comparison of topography and molecular recognition maps gives us information on interaction specificity. Force spectroscopy than allowed us to measure, and compare the strength of the binding. The presented approach can be utilized as a complementary method to classical ones such as Surface Plasmon Resonance (SPR). In addition, we employ bio-layer interferometry (BLI) measurements to complement the kinetic data of LPS *E*. *coli* B – gp37 interaction.

## Results and Discussion

According to our previous study^5^, gp37 could specifically recognize and bind only *E*. *coli* B lipopolysaccharide. However, the affinity of the binding has not been established.

**Figure 1:**
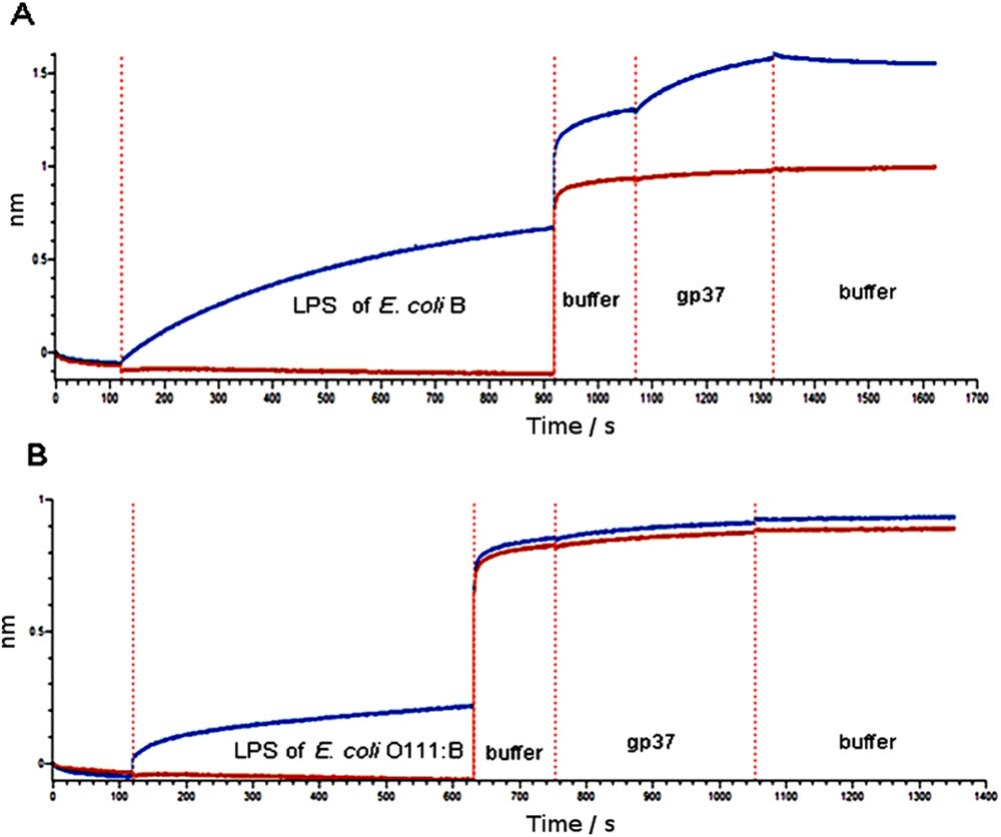
The real-time, label-free BLI analysis (based on SPR phenomenon) for PS binding to phage T4 adhesin gp37. (**A**) The positive LPS of *E. coli* B (ligand) with gp37 (analyte) interaction, (**B**) the negative LPS of *E. coli* O11:B with gp37 interaction. The red line stands as a signal without the ligand to show non-specific binding and the blue line stands as a signal of the study sample. The gp37 (667 nM) was diluted in the Buffer: PBS + 0.05%T-20 + 0.1% BSA, pH 7.4 and the LPS (50 µg/ml) was diluted in PBS, pH 7.4. The washing buffer, as well as the dilution buffer, contained additives (0.05%T-20 + 0.1% BSA) which increased the signal comparing to the PBS alone used to the LPS dilution.

In this paper, at first, we checked the binding ability of gp37 to LPS of *E. coli* O:111 and H. alvei using bio-layer interferometry (BLI) test. The purity of isolated LPSs have been analyzed in SDS-PAGE, moreover neither nucleic acids, nor protein contamination were observed in LPS specimens. The real-time BLI analysis (based on SPR phenomenon) shows strong affinity of adhesin gp37 to the LPS of *E*. *coli* B (Fig. 1A), while no interactions between gp37 and LPSs of *E*. *coli* O111:B (Fig. 1B) and *H*. *alvei* were observed. We estimated the dissociation constant (KD) between the adhesin gp37 and the LPS of *E*. *coli* B as 1.05 × 10^−7^ M, which means that the binding between the LPS and gp37 is strong. The estimation of KD for the binding pair LPS/gp37 was processed for the protein concentrations of 175, 350 and 700 nM applying global full fitting according to the 1:1 Langmuir model^14^.

Next, atomic force spectroscopy was used to investigate the interactions between the gp37 adhesin and the LPSs. Therefore we modified AFM tips with the gp37 containing 6 Histidine (His6) residues at the N-terminus. The C-terminal region of the protein mediates its specific interaction with LPS. The tip modification has been designed to use three NTA-Ni2+ groups located in such close proximity to bind all six histidine residues of the His6 tag of the adhesin^15,16^. This allowed us to obtain binding between gp37 and the surface of the tip. Moreover, this type of immobilization to the surface allowed us to expose the domain responsible for the interaction and mimic the orientation as it is in the phage particle. In its native state gp37 is a fibrous parallel homotrimer containing 1026 amino acids per monomer. It is a part of a long tail fiber in the phage particle localized in the distal part of the fiber^17^. Gp37 used in the experiment was a stable homotrimer containing the complex interweaving of the monomers (each contained 1014 amino acid residues). The protein was coexpressed together with two chaperones for correct folding and for high stability^18^. Modified tips containing immobilized gp37 were used to scan samples of LPS deposited on mica surfaces. In our experiments we used the Agilent PicoTREC mode which allowed us to record a sample topography image simultaneously with a map of interactions between the tip and the surface.

**Figure 2:**
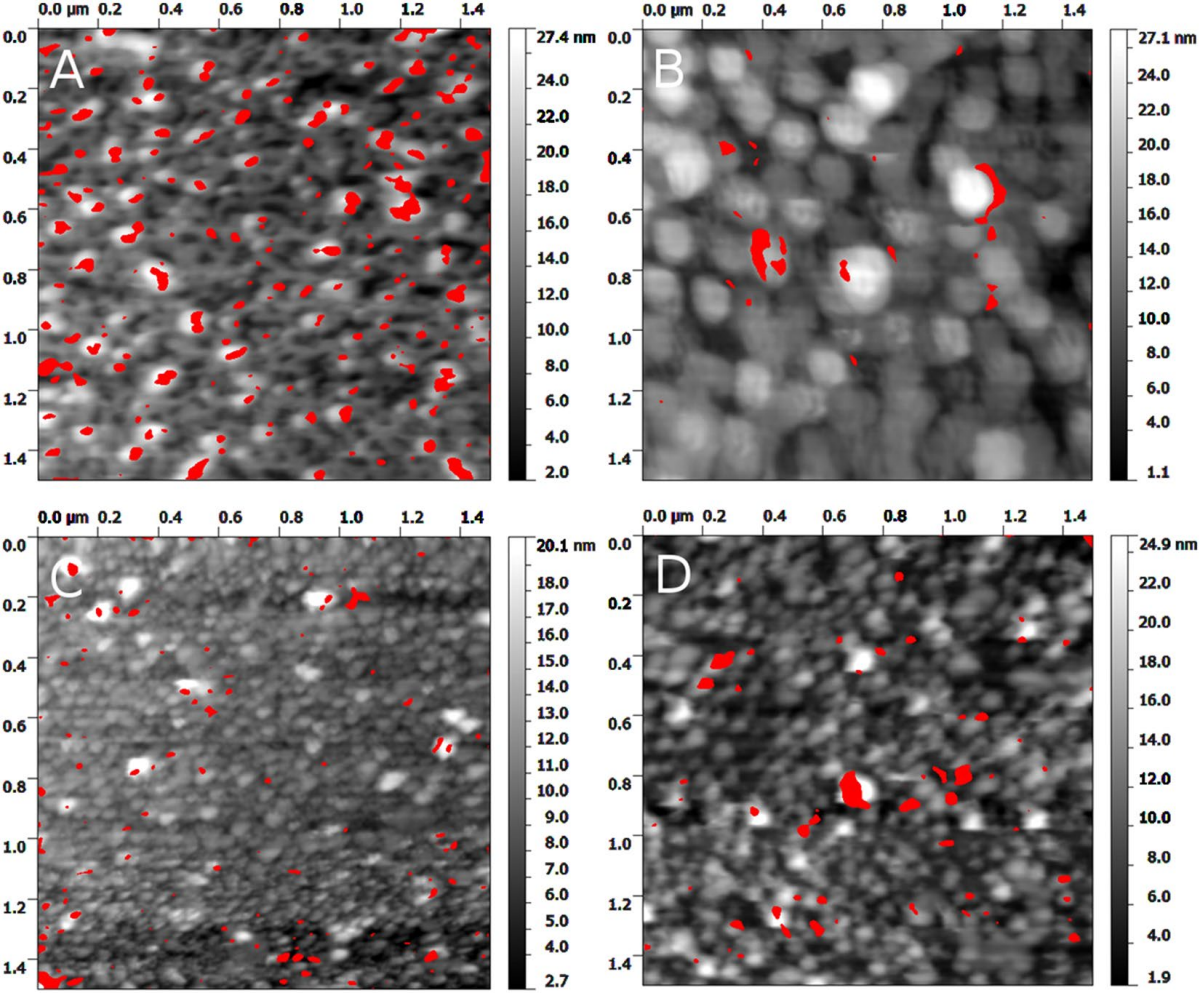
Topography image of mica modified with LPS from *E*. *coli* B (**A**,**B**); LPS from *E*. *coli* O111:B (**C**) and LPS from *H*. *alvei* (**D**) with molecular recognition images superimposed in red, showing where the value exceeds a certain threshold chosen arbitrarily to illustrate the interaction. Image B was recorded with a tip blocked with *E*. *coli* B LPS-SUV.

**Figure 3:**
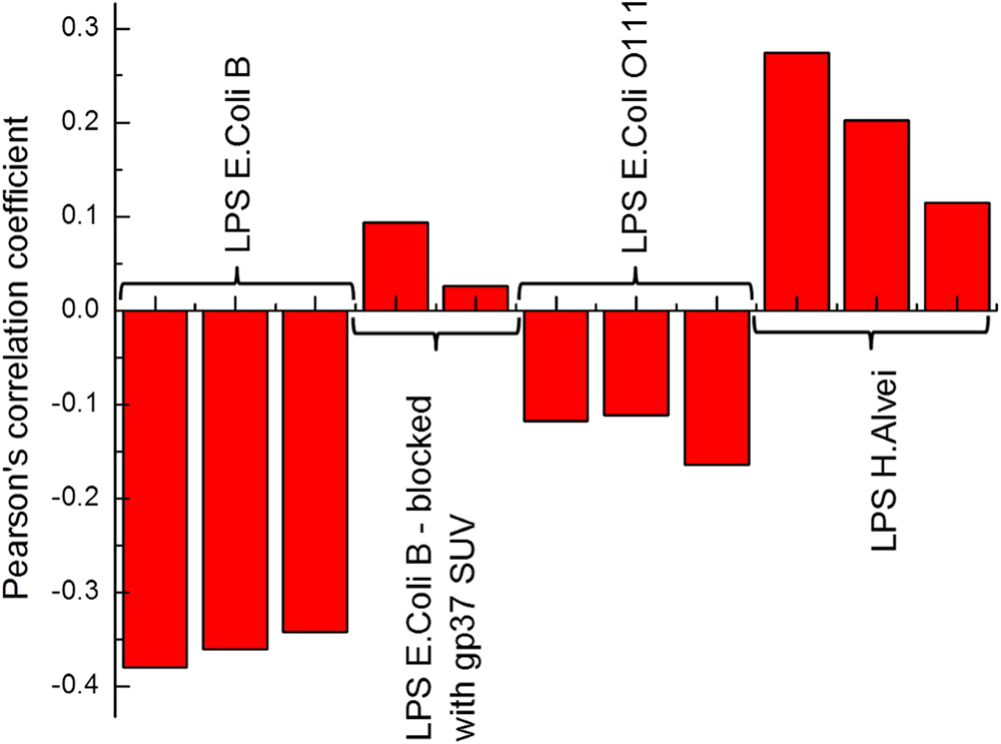
Pearson’s correlation coefficient (PCC) values for the pairs topography – molecular recognition images for each kind of the LPS.

In this technique the AFM tip modified with gp37 was oscillating over the LPS covered surface. The oscillation amplitude is dependent on the tip-surface distance. The amplitude decreases when the tip is close to the surface, due to tip-surface interaction. The tip starts interacting with the surface and the oscillation amplitude is being damped. If the tip is far enough from the surface to stretch the elastic linker connecting it with the gp37 protein the amplitude also decreases – part of the energy is used for the linker stretching. The latter effect is observed only when gp37 protein is bound to the surface. The above-mentioned phenomena influence the lower and the upper part of the amplitude sinusoid, respectively. With the help of electronics, the overall signal can be processed in a way that the information on surface topography (lower part of the sinusoid) and molecular recognition (upper part of the sinusoid) can be separated and recorded as two maps^19^. One can clearly see the correlation between surface topography and tip-surface molecular interaction for the LPS from *E*. *coli* B (Fig. 2A). The correlation disappeared when we blocked the tip with *E*. *coli* B LPS sonicated unilamellar vesicles (SUV) (10 min incubation) (Fig. 2B). We observe low correlation for LPS from *E*. *coli* O111:B (Fig. 2C), and no correlation for the LPS from *H*. *alvei* (Fig. 2D). To quantify the observed phenomena, we calculated Pearson’s correlation coefficient (PCC) for the topography/molecular recognition image pairs for each kind of LPS. Relatively strong negative correlation (PCC − 0.35 ± 0.02) (Fig. 3) were observed for LPS from *E*. *coli* B. This indicates the strong molecular interactions of gp37 adhesin with the topography features of the LPS from *E*. *coli* B. After blocking the tip with LPS from *E*. *coli* B SUV the correlation almost completely disappeared (PCC 0.06 ± 0.05). This confirms that the previously observed correlation was due to strong affinity between the LPS from *E*. *coli* B and gp37. We observe almost three times weaker negative correlation (PCC −0.13 ± 0.03) between topography and molecular recognition images for LPS from *E*. *coli* O111:B, and low positive correlation (PCC 0.20 ± 0.08) for LPS from *H*. *alvei*. The sign of the PCC indicates that the gp37 modified tip more likely interacts with the surface between the deposited LPS from *H*. *alvei* then with the LPS itself. The above described analysis confirms that the gp37 adhesin functionalized AFM tip interacts most likely with the LPS from *E*. *coli* B. In the case of the other two tested LPSs the correlations between topography and molecular recognition images were much less pronounced.

**Figure 4:**
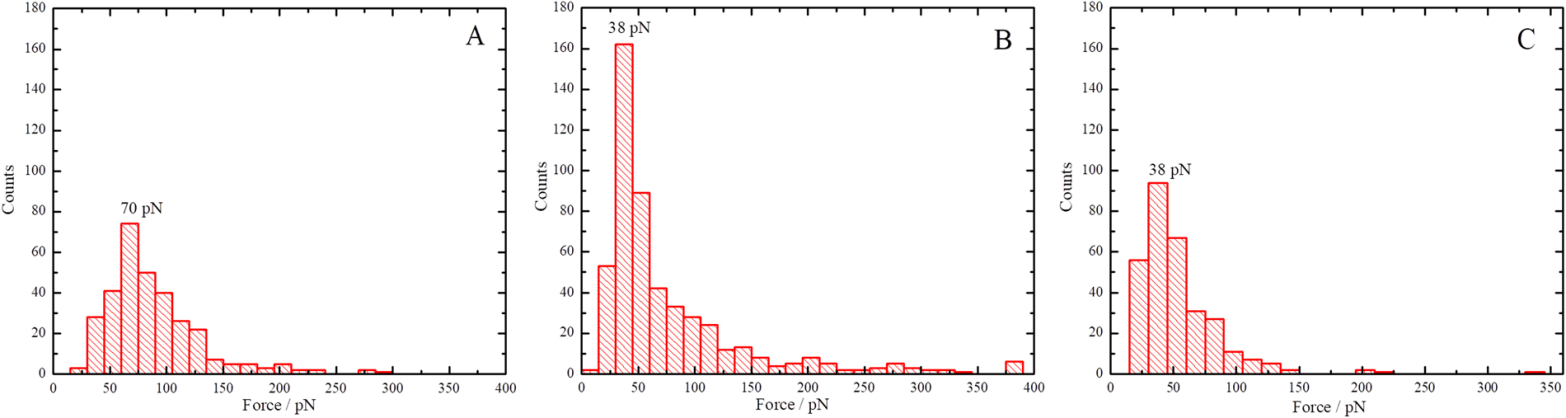
Unbinding histograms measured between gp37 adhesin modified tip and LPS from *E*. *coli* B (**A**); LPS from *E*. *coli* O111:B (**B**) and LPS from *H*. *alvei* (**C**).

To obtain quantitative data for the interaction strength between gp37 adhesin and various LPSs we performed force spectroscopy experiments. First, LPS modified mica samples were scanned with the gp37 modified tip again with the PicoTREC mode. Next, we collected force distance curves from a spot where the molecular recognition was clearly observed (Fig. 4). We recorded one thousand curves where the AFM tip approaches the surface and then retracts, while the deflection is measured in each experiment. Every LPS layer was measured in four different spots using several tips. All tips were modified according to the same procedure described below.

We analyzed all the recorded curves looking for unbinding events. To find out if the binding event had occurred, careful analysis of the “retrace” force distance curve was necessary. If the binding event had occurred, the retrace force-distance curve was showing an attractive force of the characteristic shape, which increasing nonlinearly and is associated with the stretching of the flexible linker which connects the gp37 molecule to the AFM tip. This stretching continues until a particular force - *f*_*u*_, at which the gp37–LPS complex dissociates, is achieved. Then the attractive force rapidly drops to zero, what is interpreted as the gp37 – LPS complex dissociation^20^. We present the results in the form of histograms (Fig. 4, histograms for all samples are shown in the SI). A well-developed peak at 70 ± 29 pN was observed in the histograms created for LPS from *E*. *coli* B samples. For some of the samples an additional peak at around 40 pN was observed. A peak with similar unbinding force (46 ± 13 pN) was also observed for the LPS from *E*. *coli* O111:B and from *H*. *alvei* (45 ± 14 pN) suggesting that it comes from non-specific binding events. The unspecific binding observed for all three LPSs is most probably due to gp37 protein trimer modified tip penetrating the LPS bilayer and being caught there by the van der Waals forces among the long chains of LPS molecules. This phenomenon is not related with the specific (gp37 – LPS) interaction therefore it is common for all three different LPSs. Once the protein is adsorbed on the solid surface one needs to use some force to detached it again. This phenomenon is not observed in standard biological methods, such as ELISA, because washing steps are applied to remove non-specifically adsorbed proteins from the surface. No peak at 70 pN was observed for LPS from *E*. *coli* O111:B nor for LPS from *H*. *alvei*. This kind of interaction seems to be characteristic for the pair adhesin gp37 – LPS from *E*. *coli* B. These values were obtained by fitting the histogram data with Gaussian functions and averaging the centre point and width for the different data sets.

Based on these studies we are able to establish what the physical value of the reversible binding between T4 phage and *E*. *coli* B strain is. As was mentioned earlier, gp37 adhesin is a part phage LTF participating in bacteria recognition and the reversible binding to the bacteria. At least three of the six LTFs are involved in the interaction, so we expect the minimal force necessary to break the interaction between the phage and its host should be about 210 pN (70 ± 29 pN × 3), and the maximal about 420 pN. Therefore, the measured unbinding force can also be used to determine whether the phage can continue a Brownian walk after binding or start the infection process.

## Conclusions

The paper describes the investigation of interactions between T4 phage adhesin and LPSs using AFM. The mica surface was modified by LPS and the AFM tip by T4 phage adhesin. It was demonstrated that despite all LPSs were binding to the adhesin, it is possible to distinguish between specific and nonspecific LPSs using AFM PicoTREC mode and analyzing the correlation between topography and molecular recognition images. To the best of our knowledge such an approach has not been described before. Moreover, the interaction between bacteriophage T4 adhesin gp37 and LPSs from various bacteria have been measured directly using force spectroscopy. The force necessary to break the interaction between gp37 and LPS from *E*. *coli* B, LPS from *E*. *coli* O111:B and LPS from *H*. *alvei* were measured to be 70 ± 29 pN, 46 ± 13 pN pN and 45 ± 14 pN, respectively, where the latter vales are due to interaction with the sample surface. The proposed methodology gives us an information about the physical position of the interacting LPS. By comparing the topography and recognition maps one can calculate the correlation between them. The correlation coefficient gives us information on the interaction specificity. Moreover, the independent, mechanical force based, binding strength measurement can be beneficial in eliminating the errors that can happen if only one method is applied. The data will be useful for biosensor development for pathogenic bacteria and endotoxins detection.

## Experimental Section

### LPS extraction and purification

Bacterial *E*. *coli* B (PCM 1935) and *Hafnia alvei* (PCM 1189) strains used in the experiments were obtained from the Polish Collection of Microorganisms (PCM) of the Hirszfeld Institute of Immunology and Experimental Therapy, PAS in Wroclaw, Poland. LPSs were extracted from dry mass of bacterial cells using the hot phenol-water method as described previously by Westphal and Jann^21^. The collected extracts (aqueous phases) were dialyzed against distilled water, concentrated, and treated with ethanol, to obtain an 80% ethanol solution. The precipitated LPS was dissolved in phosphate-buffer saline (PBS) buffer pH 7.0 and treated with DNAse and RNAse (0.01 mg/ml for 4 hours at 37 °C). At the end, the Proteinase K was added for two more hours. Finally, the LPS was collected as a precipitate in ultracentrifuge by pelleting it at 40000 rpm for 6 hours. The ultracentrifugation was repeated four times. The LPS was re-suspended in water and lyophilized. The LPS of *E*. *coli* O111:B was purchased from Sigma-Aldrich.

### Bacteriophage T4 dhesin gp37 purification

The recombinant phage T4 dhesin gp37 was prepared according to the methods reported previously by Brzozowska *et al*.^5^.

### Monitoring of LPSs real-time binding using BLI

The binding assays between LPSs and gp37 phage adhesin were performed using an Octet RED96 system (Pall ForteBio, Fremont, CA, USA). The LPS (ligand) was immobilized to the Aminopropylsilane Sensors (APS) in PBS, pH 7.4. The kinetic measurements were carried out by exposing sensors with serially diluted gp37 adhesin in a binding buffer (1× PBS, pH 7.4, containing 0.1% BSA and 0.05% Tween-20) in the wells of a 96-well microtiter plate. The association and dissociation profiles of the compounds were monitored at 30 °C. Binding curves were analyzed by global fitting of sensorgrams to 1:1 model using Octet software provided by Pall ForteBio. The concentration of the ligands – LPS was 50 μg/mL and the concentration of the analyte – gp37: 667 nM. Analyzed concentrations taken for KD calculations were: 175, 350 and 700 nM.

### AFM measurements

The atomic force spectroscopy measurements were done with a 5500 AFM device (Keysight Technologies). The data was analyzed with dedicated software. The LPS bilayers were deposited on mica surface and the AFM tip was modified with gp37 protein. The measurements were performed with MAC Lever type VII cantilevers (Keysight Technologies) with nominal force constant k = 0.14 N/m. However, the exact value of the force constant was determined based on the thermal tune method. The tips were functionalized with a modified version of the procedure reported by Gruber^15^ using the interactions between multivalent N-nitrilotriacetic acid complexes with His-tagged proteins^16,22,23^. Briefly; the probes were washed by immersing them in chloroform (3 × 5 min on a shaker) and incubated in a desiccator for 2 hours under argon atmosphere together with 30 µl of aminopropyltriethoxysilane (APTES) and 10 µl of triethylamine (TEA). Next, APTES and TEA were removed and the probes were left for curing for 48 hours under argon. Functionalization of amino groups was performed by incubation for 2 hours in solution prepared by dissolution of 1 mg Maleimide-PEG-NHS and 30 µl of TEA in 0.5 ml of chloroform. Next, the probes were washed with chloroform, dried with argon and placed on Parafilm in a polystyrene Petri dish. To perform the next step of functionalization, the following components were premixed: 100 µl disulfide-tris-NTA (1 mM) in water; 2 µl EDTA (100 mM, pH = 7.5); 5 µl HEPES (1 M, pH = 7.5); 2 µl TCEP hydrochloride (100 mM) and 2.5 µl HEPES (1 M, pH = 9.6). The mixture was pipetted onto the AFM probes and left for 4 hours to react. After that, the probes were washed 3 times for 5 minutes with Hepes buffer (5 mM HEPES, 25 mM KCl, pH = 7.4) and 100 µl of 0.5 µM His_6_-tagged gp37 was mixed with 4 µl NiCl_2_ (5 mM) and pipetted onto the probes. The gp37 concentration selected to be high enough to be able to observe unbinding events, but low enough to eliminate simultaneous multi bond breakage. The probes were incubated for 1.5 hours and washed with HEPES buffer again. The modified probes were kept in 4 °C immersed in 5 mM HEPES buffer, 25 mM KCl (pH = 7.4).

### Preparation of LPS bilayers on mica

The mica surface was covered with LPS bilayer using a modification of the procedure described by Handa *et al*.^24^. Briefly, to obtain multilamellar vesicles (MLV) 1 mg of LPS was dissolved in 1 ml 5 mM HEPES buffer, 25 mM KCl (pH = 7.4). The mixture was incubated for 1 h in 60 °C. To obtain LPS-SUV the MLV suspension was sonicated with an ultrasonic tip (10 cycles). Each cycle contains 2 min of sonication and 2 min of standby time. After the sonication the sample was centrifuged (30 min, 14000 rpm) and the precipitate was discarded. The resulting SUV suspension was stored in 4 °C. Fresh mica surfaces were obtained by cleaving mica using a piece of scotch tape. Next, the mica was immersed into 100 ppm polyethyleneimine (PEI) for 30 min and then the surface was washed with water (3 × 5 min) in an ultrasonic bath. After the above mentioned premodification, 150 µl of the SUV suspension was deposited on the surface and incubated for 3 h in 60 °C. The sample was covered to prevent water evaporation. Finally, the surface was rinsed with 5 mM HEPES buffer, 25 mM KCl (pH = 7.4).

### Gp37 – LPS interaction measurements. AFM experiment

The interaction between gp37 adhesin and LPSs was measured with an AFM working in PicoTREC mode using the tips modified according to above described protocol. The resonance frequency of the cantilevers in solution was in the range of 12–20 kHz. The amplitude of tip oscillation during PicoTREC imaging was kept at 12 nm. The topography and molecular recognition images have been recorded simultaneously. Next, the force spectroscopy experiment were performed. In every experiment 1000 force curves were recorded. The experiment was performed in a spot where a molecular recognition signal could clearly be seen. Unbinding events were identified as steps in the force curves. The results of every single experiment were summarized in the form of a histogram. From the resulting histogram an unbinding force value was determined.

### Pearson’s correlation coefficient calculations

The Pearson’s correlation coefficients have been calculated with ImageJ image analysis software^25^ using the JACoP plugin^26^.

### Data availability statement

The authors declare that materials, data and associated protocols promptly available to readers without undue qualifications in material transfer agreements.

## Electronic supplementary material

Supplementary Dataset
