# Supplementary material for: Interactions of bacteriophage T4 adhesin with selected lipopolysaccharides studied using atomic force microscopy
Source: Sci Rep. 2018 Jul 19;8:10935. doi: 10.1038/s41598-018-29383-w (PMC6053362; doi:10.1038/s41598-018-29383-w)
Supplement: Supplementary file 1 — Supplementary Dataset [file 41598_2018_29383_MOESM1_ESM.docx]

**Supplementary materials**

**Interactions of bacteriophage T4 adhesin with selected lipopolysaccharides studied using atomic force microscopy**

Ewa Brzozowska^1*^ Adam Leśniewski^2**^, Sławomir Sęk^3^, Ralph Wieneke^4^, Robert Tampé^4^, Sabina Górska^1^, Martin Jönsson-Niedziółka^2^ and Joanna Niedziółka-Jönsson^2***^

^1^Hirszfeld Institute of Immunology and Experimental Therapy, Polish Academy of Sciences,. 12 R. Weigl,53-114 Wrocław, Poland

^2^Institute of Physical Chemistry, Polish Academy of Sciences, 44/52 Kasprzaka, 01-224 Warszawa, Poland

## ^3^Biological and Chemical Research Centre, University of Warsaw, 101 Żwirki i Wigury 02-089 Warszawa, Poland

**^4^**Institute of Biochemistry, Biocenter, Goethe University Frankfurt, Max-von-Laue-Str. 9, 60438 Frankfurt am Main, Germany

Histograms for all the measured samples.

1. LPS from *E. coli* B


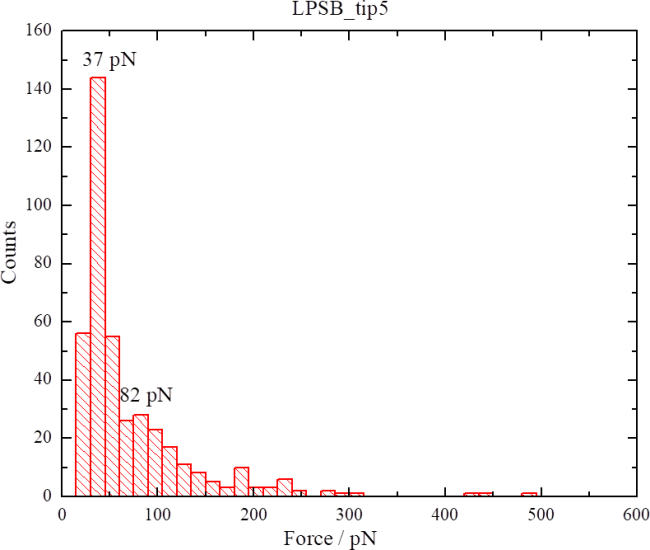

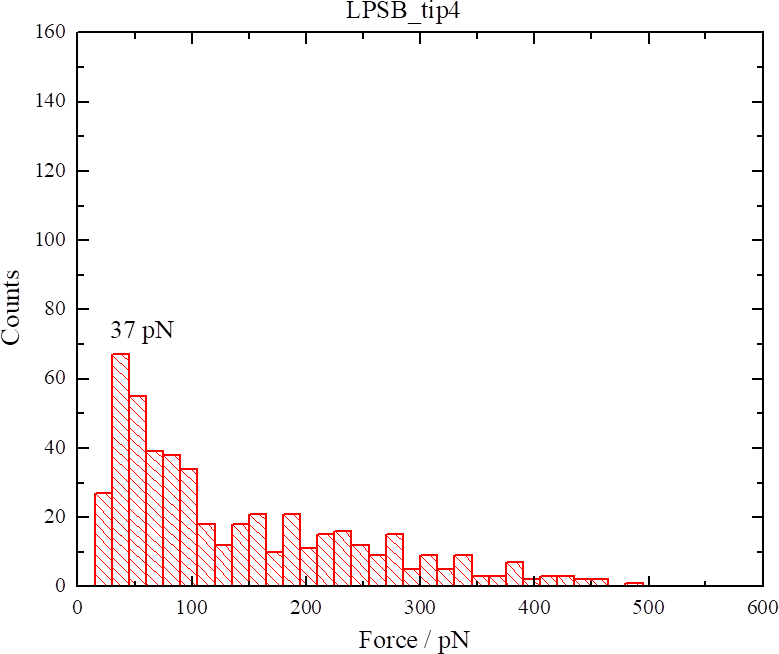

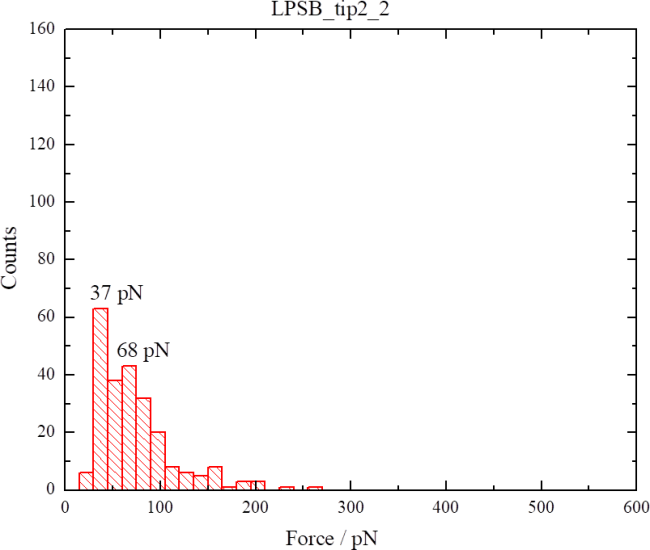

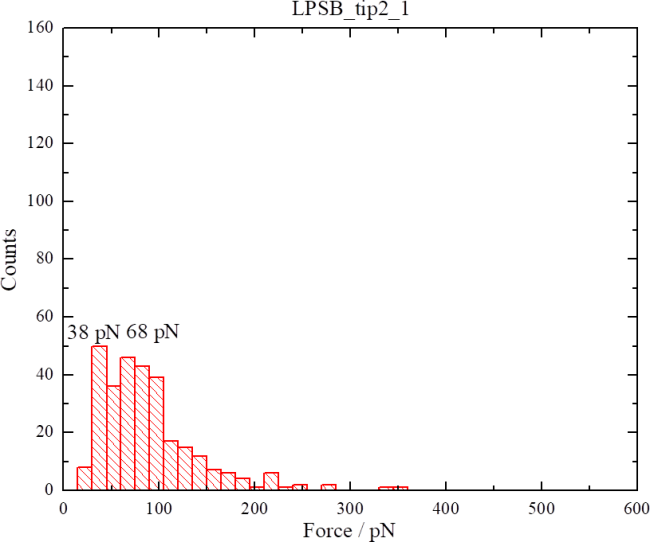

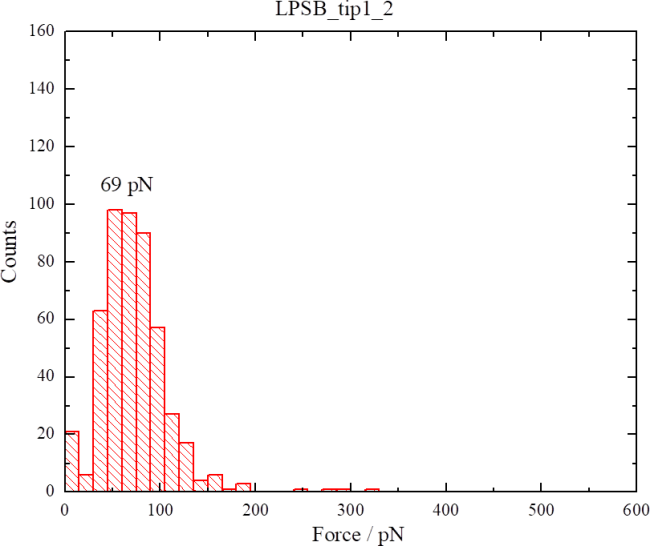

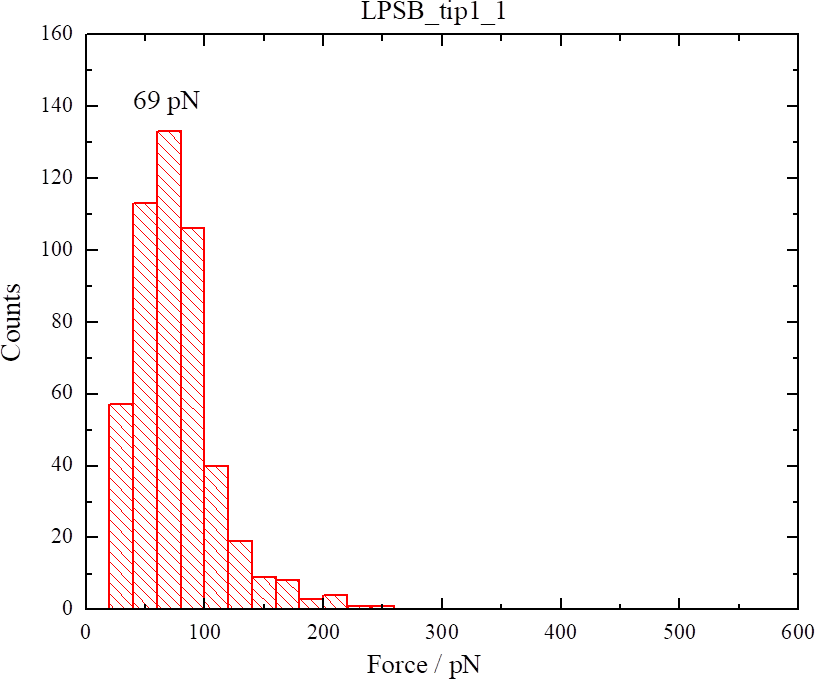

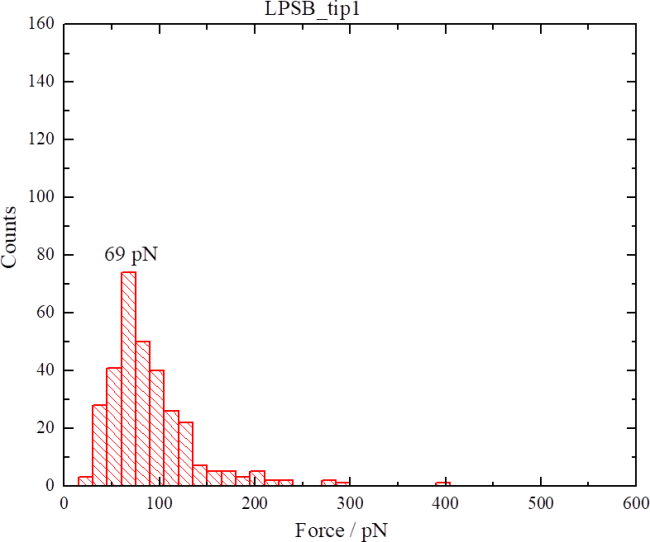


1. LPS from *E. coli* O111:B


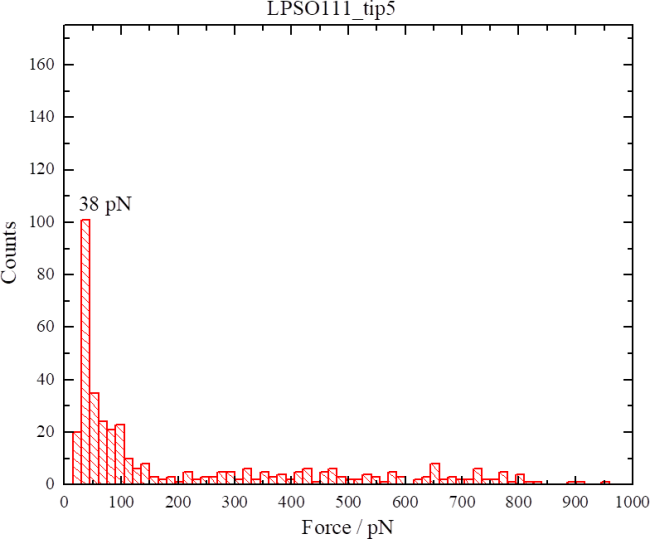

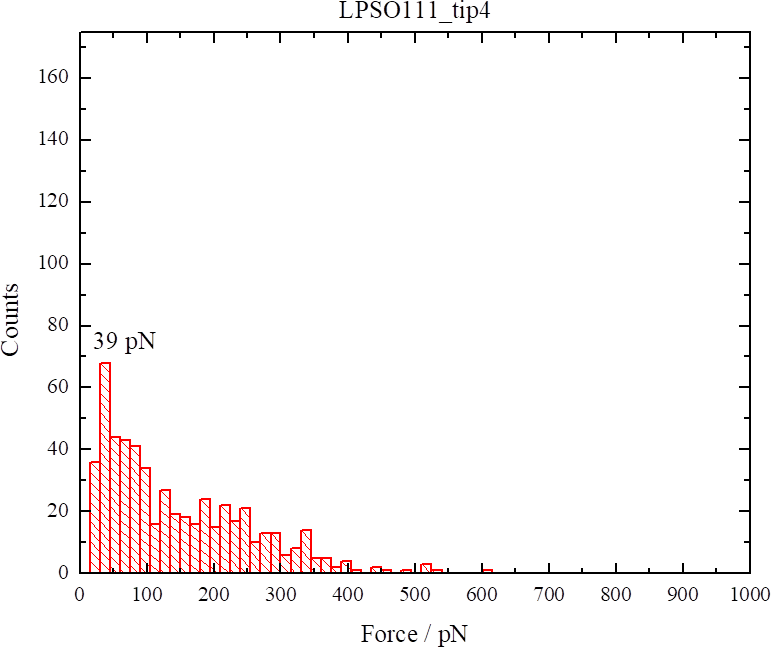

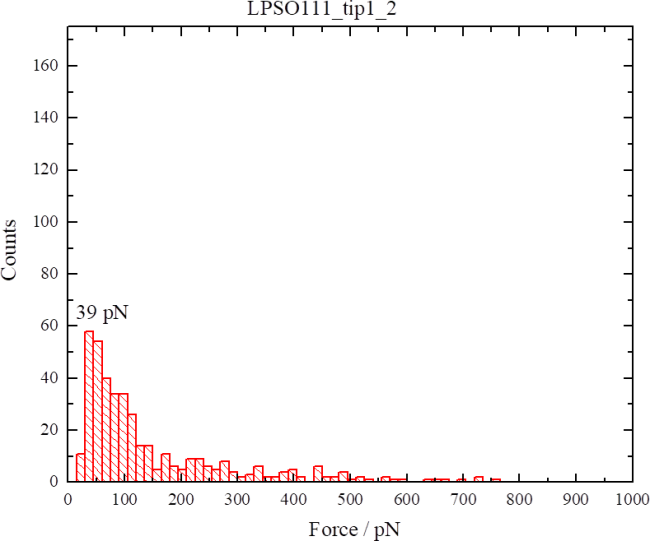

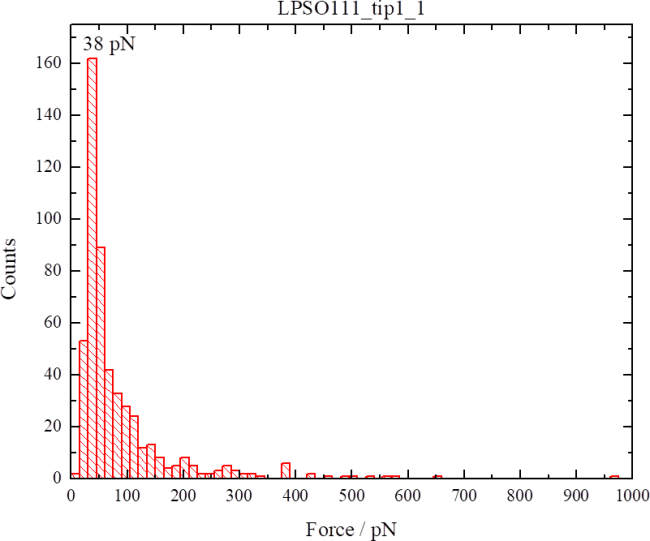

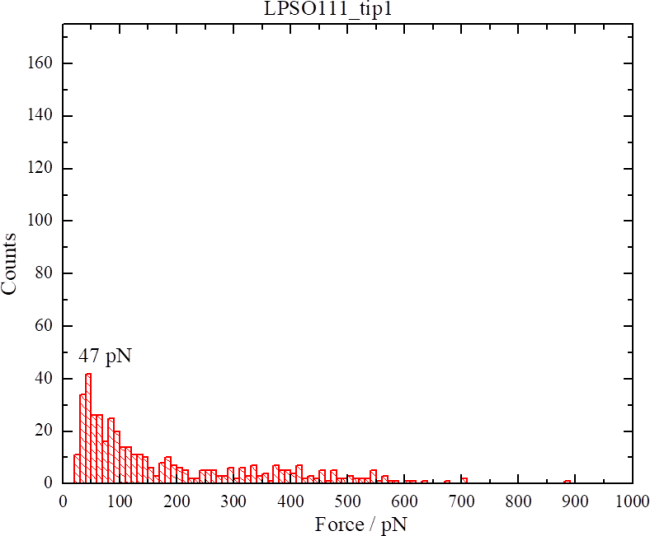


1. LPS from *H. alvei*


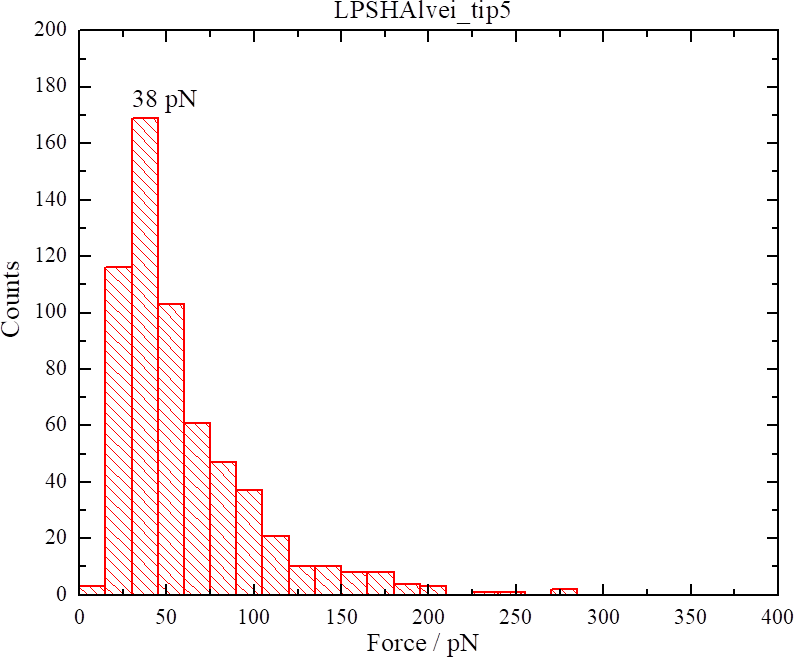

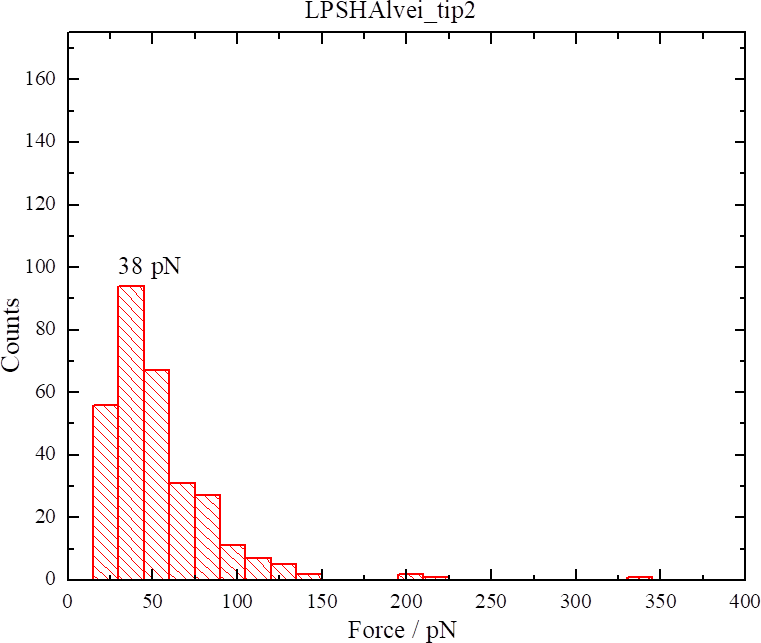

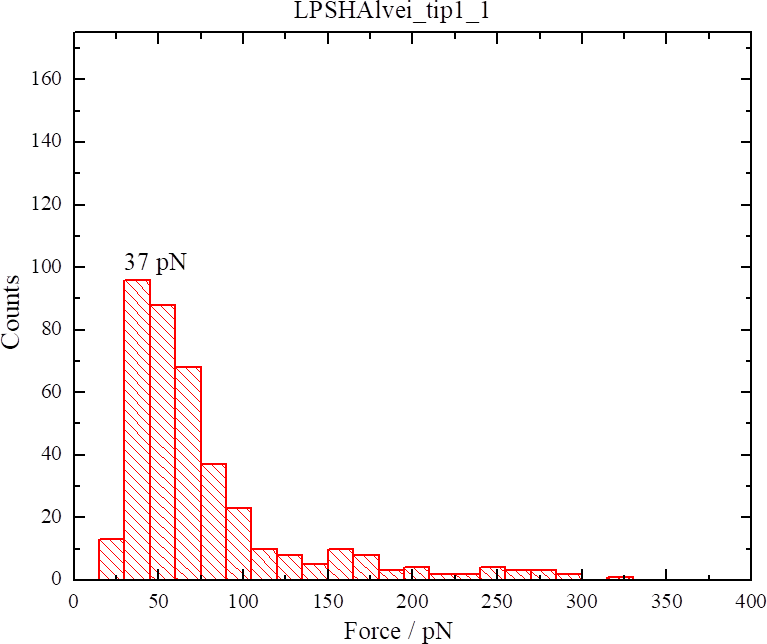

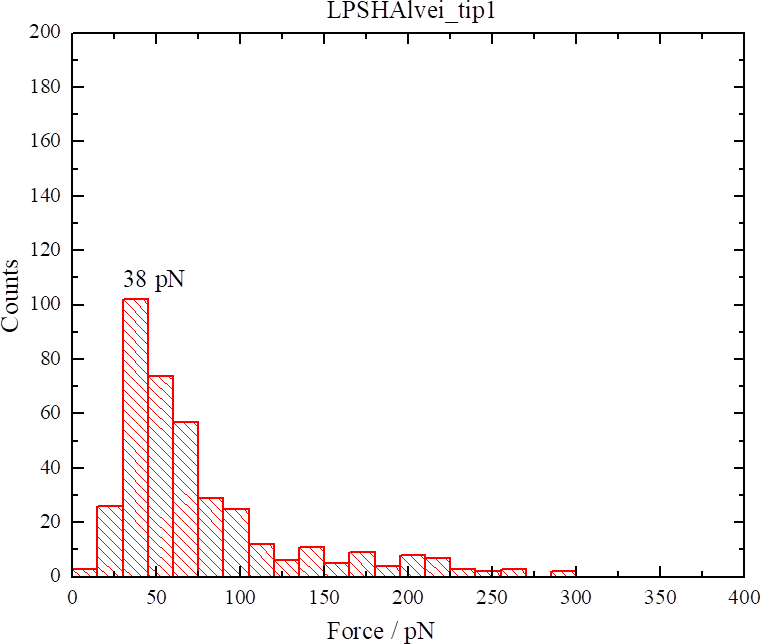


Typical force curve showing two steps, one non-specific event and one higher force specific unbinding event.
